# Implications of Probiotics on the Maternal-Neonatal Interface: Gut Microbiota, Immunomodulation, and Autoimmunity

**DOI:** 10.3389/fimmu.2018.02840

**Published:** 2018-12-03

**Authors:** Brianna Swartwout, Xin M. Luo

**Affiliations:** ^1^Translational Biology, Medicine, and Health Graduate Program, Virginia Tech Carilion Research Institute, Virginia Tech, Roanoke, VA, United States; ^2^Department of Biomedical Sciences and Pathobiology, Virginia-Maryland College of Veterinary Medicine, Virginia Tech, Blacksburg, VA, United States

**Keywords:** probiotics, gut microbiota, maternal, neonatal, autoimmunity

## Abstract

Probiotics are being investigated for the treatment of autoimmune disease by re-balancing dysbiosis induced changes in the immune system. Pregnancy is a health concern surrounding autoimmune disease, both for the mother and her child. Probiotics for maternity are emerging on the market and have gained significant momentum in the literature. Thus far, evidence supports that probiotics alter the structure of the normal microbiota and the microbiota changes significantly during pregnancy. The interaction between probiotics-induced changes and normal changes during pregnancy is poorly understood. Furthermore, there is emerging evidence that the maternal gut microbiota influences the microbiota of offspring, leading to questions on how maternal probiotics may influence the health of neonates. Underpinning the development and balance of the immune system, the microbiota, especially that of the gut, is significantly important, and dysbiosis is an agent of immune dysregulation and autoimmunity. However, few studies exist on the implications of maternal probiotics for the outcome of pregnancy in autoimmune disease. Is it helpful or harmful for mother with autoimmune disease to take probiotics, and would this be protective or pathogenic for her child? Controversy surrounds whether probiotics administered maternally or during infancy are healthful for allergic disease, and their use for autoimmunity is relatively unexplored. This review aims to discuss the use of maternal probiotics in health and autoimmune disease and to investigate their immunomodulatory properties.

## Introduction

Probiotic based therapies are emerging in modern medicine and have turned the heads of multiple large pharmaceutical companies to treat diseases associated with gut dysbiosis including colorectal cancer, multiple sclerosis (MS), inflammatory bowel disease (IBD), rheumatoid arthritis (RA), systemic lupus erythematosus (SLE), and obesity (1–4). Probiotics provide the potential to compensate for the pathological problems of gut dysbiosis and restore balance to the microbiota of the intestinal tract (5).

The definition of a “probiotic” was formed at The Lactic Acid Bacteria Industrial Platform in 1988 (6), acknowledged in joint consultation of the World Health Organization and Food and Agriculture Organization (7), and reapproved in 2014 by a panel of scientist in the International Scientific Association for Probiotics and Prebiotics (8). It resounds throughout literature as an organism that, when delivered in an adequate dose, confers a health benefit beyond normally acquired nutrition (1, 2, 9). Since the discovery of probiotics, a whole slew of healthy gut-promoting microbe-associated products are being investigated that fall under the umbrella term “pharmabiotics.” Besides probiotics, pharmabiotics include prebiotics, synbiotics, and metabolites (1). Another non-living pharmabiotic has recently been described as “paraprobiotic” or “ghost probiotic.” Ghost probiotics are comprised of dead cells and bacterial metabolites and named for the interaction with living organisms of non-living material that was once a living microbe (10, 11).

This review does not focus on all of the aforementioned probiotic related products, but galvanizing research is being conducted on how metabolites from probiotics can confer protective effects upon their host. Recently, *Lactobacillus acidophilus* bacteriocin was proven to inhibit the growth of opportunistic pathogens *Gardnerella vaginalis, Streptococcus agalactiae*, and *Pseudomonas aeruginosa* which are also commensal (12). Metabolites isolated from cultured *Bacillus coagulans* drove maturation of monocytes (13). Short chain fatty acids, like butyrate, are another metabolic derivative of probiotics that are implicated in reducing disease severity in cancer and autoimmunity (14, 15). Butyrate has been shown to regulate the diversity of bacteria species in the gut as well as improve the function of the intestinal epithelial layer (16). Cell surface components and supernatant containing bacterial metabolites differentially stimulate immune cells by prompting either pro- or anti-inflammatory cytokine production (17). Therefore, metabolites perform a variety of beneficial functions including antimicrobial activity, enhancing the gut barrier, modulating microbiota diversity, and immunomodulation, all of which are implicated in the amelioration of autoimmune disorders. A detailed discussion on how bacterial metabolites may affect health and autoimmune diseases can be found elsewhere (14, 18).

Current clinical trials involving probiotics include using pet dogs to modulate the microbiota in a probiotic capacity in the elderly, prevention of necrotizing enterocolitis in preterm infants, and intervention for anxiety and depression (19–21). The only clinical trials investigating probiotics in autoimmunity are two studies on type 1 diabetes (22, 23), and one study on IgA Nephropathy (24). While the benefits of probiotics for intervention of gut dysbiosis are established, all angles of probiotic use need to be closely examined. Here we review probiotics influence on the gut microbiota, immune system, and autoimmunity in the normal, maternal, and fetal context.

## Probiotics

### Changes to gut microbiota

Ten trillions of microbes inhabit the human gastrointestinal tract, the majority of which are bacteria (25). Difference in the gut microbiota occurs throughout the gastrointestinal tract. From the oral cavity to the colon, the microbiota changes. The esophagus, duodenum, and jejunum contain *Streptococcus* as the predominant genus, whereas the stomach is colonized by *Streptococcus* and *Prevotella* (25, 26). However, individuals who are carriers for the species *Helicobacter pylori* in their stomach have more bacteria from the phylum Proteobacteria (25, 26). The colon contains the richest diversity and highest abundance of bacteria, the bulk of which are from the phyla Firmicutes and Bacteroidetes (25, 26). Firmicutes is mostly comprised of gram positive species from several notable genera like *Enterococcus, Helicobacterium, Streptococcus, Staphylococcus, Lactobacillus, Clostridium, Lachnospireceae*, and *Streptococcus* (27). The phylum Bacteroidetes is comprised of gram negative rods, of which the most notable genera found in the gastrointestinal tract are *Bacteroides* and *Prevotella* (28). Differences in microbial populations also exist between the lumen and epithelium. A study using fluorescent *in situ* hybridization on sections of the colon showed that from the epithelium to the lumen the diversity of bacteria increased (26, 29). In the distal colon, a layer of interlaced bacteria barred the mucosa from *Bacteroides* and *Clostridium difficile* (26, 29). No bacterial probes hybridized with the mucosa itself, but bacteria present in the crypts—*Lactobacillus, Coriobacterium, Phascolarcotbacterium, Clostridium*, and assorted *Proteobacteria* genera—were also present in the interlaced layer and luminal content (26, 29). All of the bacteria in the interlaced layer, which included all those found in crypts as well as more species of *Clostridium* and some *Bacteroides*, were also found in the lumen (26, 29). The luminal content contained the highest number of different bacteria and included prominent genera such as *Bifidobacterium* and *Enterobacteriaceae* (26, 29). Other techniques of analyzing mucosa-associated microbes include washing away the luminal content and scraping the mucosa under sterile conditions or do not include the cross-sectional refinement of the aforementioned study (30, 31).

As an organ, the health of normal gut microbiota can be enhanced by probiotics. Probiotics have the capacity to antagonize harmful bacteria in the gut by secretion of antimicrobials, competitive adherence to the mucosa, strengthening of the epithelial layer, and modulation of the immune system (9). Probiotics have been shown to stabilize bacterial populations during changes in diet (32) and at the same time change the architecture of the gut microbiota (33). Probiotic mixtures of *Lactobacillus, Bifidobacterium*, and *Streptococcus* species alter the landscape of the gut microbiota by increasing the number of Firmicutes and Actinobacteria while decreasing the number of Bacteroidetes and Proteobacteria (33–35). This same shift in bacterial populations is seen both in healthy controls as well as diseased individuals (36). Probiotics increase the number of *Lactobacilli* and *Bifidobacteria* secreted in feces, indicating their capacity for colonization of the human gut, which can be enhanced by coating the bacteria in a capsule that protects them from the gastroduodenal environment (37). In colorectal cancer patients, treatment with *Lactobacillus acidophilus, Bifidobacterium longum*, and *Enterococcus faecalis* decreased the abundance of pathogenic *Fusobacterium*, the prevalence of which is associated with both IBD and colorectal cancer (2). Besides influencing bacterial populations of the gut, probiotics may also ameliorate disease-associated imbalances in the fungal microbiota. A recently published study showed a probiotic mixture of *Lactobacillus rhamnosus* and *Bifidobacterium animalis* decreased serum IgG levels against *Candida albicans* in schizophrenia associated dysbiotic conditions (38).

Most studies with probiotics involve using them either on patients or animal models of disease. The challenge is to delineate how different species and genera impact the normal gut microbiota. For example, treatment of obese mice on a high fat diet with two strains of *Lactobacillus, L. curvatus* and *L. plantarum*, changed the microbiota by increasing *Lactobacillus* species, Clostridiales including Ruminococcaceae and Lachnospiraceae, and *Bifidobacterium* (39). Other studies in mice found that treatment with *L. acidophilus* may have decreased the abundance of *Lactobacillus* (40). Increases of Lachnospiraceae with *Lactobacillus* treatment was supported in a study on *L. rhamnosus* GG (LGG) treatment of patients with cirrhosis that found increases in Lachnospiraceae and Incertae sedis Clostridia XIV, while the abundance of Enterobacteriaceae and Porphyromonadaceae decreased (41). This study did not describe any changes to Lactobacillaceae. Intervention with *Bifidobacterium bifidum* at the phylum level increased the ratio of Firmicutes to Bacteroidetes, and at the genus level decreased *Bacteroides, Faecalibacterium*, and *Lachnospira* with increasing *Ruminococcus, Dorea*, and *Streptococcus* (42). Interesting, directly post intervention there was a transient rise in *Bacteroides* and *Lachnospira* before the abundance decreased. Thus, while it is difficult to account for difference in dose and disease, probiotic *Lactobacillus* and *Bifidobacterium* differentially alter the gut microbiota leading to the conclusion that selection of probiotics must consider the pathology of dysbiosis.

### Modulation of immune system

Most probiotic strains of bacteria are gram positive bacteria, meaning that they will interact with Toll-like receptor (TLR)-2, responsible for recognition of bacterial peptidoglycan, and produce pro-inflammatory cytokines, such as IL-6 (43, 44). Several studies have shown that TLR2 is upregulated in macrophages and epithelial cells after treatment with probiotic species (45–47). Paradoxically, TLR2 stimulation is known to activate NF-kB through a tyrosine kinase dependent manner (48), but NF-kB signaling, along with resulting pro-inflammatory cytokine expression, has been shown to be downregulated with probiotic treatment with these bacteria (49, 50). Ryu et al. showed that, in mice, pathological consequences of *C. rodentium* infection is decreased by pretreatment with LGG. Although the study showed that histological and mortality was not improved in TLR2 knockout (KO) or TLR4 KO, pro-inflammatory TNF-α, IFN-γ, and MCP-1 were downregulated in the TLR4 KO, but not the TLR2 KO (51). Therefore, down regulation of pro-inflammatory cytokine production resultant from activation of NF-kB may be dependent on TLR2 stimulation. Actually, probiotic treatment upregulates anti-inflammatory molecules such as IL-10 and TGF-β and downregulates pro-inflammatory molecules such as IL-8, IL-1β, and TNF-α (46, 52). Yet, probiotics do not completely truncate pro-inflammatory signaling as upregulation of IFN-γ and IL-2 was observed in the Peyer's patches of mice treated with *L. casei* supplemented yogurt (53), and they have been shown to enhance phagocytic activity of granulocytes and monocytes (54). Taken together, these studies suggest that the anti-inflammatory effects of probiotics may be due the development of immune tolerance by a refined modulation of pattern recognition receptor (PRR) activation or by another mechanism altogether.

Probiotics also modulate the adaptive immune system by increasing the ratio of regulatory to effector T cell populations (52, 55–58). One putative mechanism of increasing T regulatory (Treg) cells is through inducing regulatory dendritic cells (DCs), which were determined via co-culture by Kwon et al. to cause differentiation of T cells into inducible Treg (iTreg) cells in peripheral tissue by upregulating forkhead box P3 (Foxp3) (52, 56, 59). The upregulation of Treg cells occurs concomitant with suppression of effectors cells such as T-helper (Th)1, Th2, and Th17 (60). Imbalances in these populations associated with disease are restored by probiotics (55, 61). Furthermore, some probiotics beneficially enhance B cell responses. Asthma patients treated with *Clostridium butyricum* enhanced immunotherapy by improving serum-specific IgE, increasing IL-10 producing B cells, and enhancing antigen specificity in peripheral blood B cells (62). A strain of heat killed *L. paracasei* isolated from a human adult induced increases in antigen-specific IgA production in mice as well as increases in T follicular helper (Tfh) cells (63). *L. plantarum* strain AYA was similarly found to increase differentiation of IgA producing B cells into plasma cells resulting in increased IgA production in the small intestine and lung, thus providing protection against infections (64). Altogether, probiotics induce regulation of the immune system by promoting shifts in lymphocyte populations and promotion of an anti-inflammatory environment.

### Effect on autoimmunity

Autoimmune disorders are believed to result from genetic predisposition and interactions with the environment (65, 66). Dysregulation of commensal microbial communities and interactions with pathogens are an emerging hallmark of autoimmune disease (67). Many autoimmune disorders have recently been associated with gut dysbiosis including SLE, type 1 diabetes, RA, and MS (68–71). Some autoimmune disorders are thought to be catalyzed through molecular mimicry by microbes. For example, 50–70% of cases of Guillain-Barré syndrome, an autoimmune condition and complication of Zika virus infection, occur after either a respiratory or gastrointestinal infection (72). Instances of Guillain-Barré have been associated with Zika virus, *Campylobacter jejuni*, cytomegalovirus, Epstein-Barr virus, measles, influenza A, and *Mycoplasma pneumoniae* (72). Acute rheumatic fever, another autoimmune driven illness, occurs after *Streptococcus pyogenes* infection (66). Arthritis has also been associated with infection of gut pathogens such as *Shigella, Salmonella, Yersinia*, and *Campylobacter* and synovial fluid has been found to contain antigens from these pathogens (73, 74).

Disrupting autoimmune disorders through probiotic intervention has generated a lot of interest as a healthcare strategy for chronic autoimmune disorders such as SLE (75). Our lab has shown that *Lactobacillus* species can beneficially impact the progression of glomerulonephritis, an inflammatory kidney syndrome occurring in over half of all SLE patients, in MRL/lpr mice (61). Other labs have shown the beneficial effects of probiotics on SLE in NZB/W F1 mice, another classical SLE mouse model (76, 77). Cardiac cell apoptosis in NZB/W F1 mice was attenuated by *L. paracasei* (76) and hepatic cell apoptosis was ameliorated by mixture of *L. paracasei* and two different strains of *L. reuteri* (77). Several studies have shown that probiotics may have a protective effect against respiratory infections (78, 79), which may mitigate the potential for those autoimmune disorders caused by molecular mimicry. At the frontier of probiotic research, ghost probiotics are being investigated for their potential to protect from infections associated with autoimmune disorders (10, 11).

One role of probiotics in dampening autoimmunity is through enhancing gut barrier function. Leaky gut, a condition characterized by a disrupted epithelial layer, is caused by loosening of the tight junction proteins that hold a contiguous barrier in the gastrointestinal tract, and results in penetration of foreign antigens and harmful substances (80). This condition can arise from diet and stress induced dysbiotic conditions and excessive inflammation (81, 82). In autoimmune disease, a leaky gut can be either etiological or aggravating (80), and is characteristic in mouse models and humans with autoimmune disease (61, 83). Pathobionts like *E. gallinarum* translocate into non-gut tissues under leaky gut conditions and exacerbate disease in a lupus mouse model (NZWxBXSB) (83). We have previously shown that *Lactobacillus* species can effectively improve the intestinal barrier of a different strain of lupus-prone mice, MRL/lpr (61). The capacity for probiotics, prebiotics, and metabolites to enhance gut epithelial barrier function is an indication for their effectiveness in treating autoimmune diseases (61, 70).

Probiotics have been shown to be beneficial in a number of autoimmune diseases. In SLE, the ratio of Th17 to Treg cells is increased (84), but can be restored by probiotic mixture of *Lactobacillus* species (61). In a clinical trial, treatment with *L. casei* significantly decreased TNF-α and IL-10 in patients with RA (85). Although IL-12 significantly decreased in both the control and probiotic group, the ratio of IL-10 to IL-12 at the end of the study was significantly higher in the probiotic group (85). The clinical course of MS is improved by shifting a Th1 response to Th2 and, among drugs such as glatiramer acetate, probiotics also have the potential for this particular immunomodulation (86). For example, a phase I clinical trial using probiotic helminths has shown increases in IL-4 and IL-10, Th2 signature cytokines (87). Experimental models of MS also showed promising immunomodulatory effects of a mixture of probiotics called IRT5 (*L. casei, L. acidophilus, L. reuteri, B. bifidum, Streptococcus thermophiles*) by balancing Treg to the effector Th1 and Th17 cells (88). Early probiotics have also been associated with protection against type 1 diabetes islet autoimmunity in an international longitudinal observational study on children at risk (89). Based on these evidences, probiotics may serve as a means of correcting the effects of autoimmune-related dysbiosis and promoting the health of patients suffering from autoimmune disorders or diseases. However, there is little research investigating the impact of maternally administered probiotics on the development of autoimmunity in mothers and infants.

## Maternal probiotics

### Pregnancy induces changes in gut microbiota

The normal microbiota undergoes changes during pregnancy including increases in oral presence of *Porphyromonas gingivalis, Aggregatibacter actinomycetemcomitans*, and *Candida*; anaerobic and aerobic bacteria increase in the placenta; the gastrointestinal tract increases in Actinobacteria, Proteobacteria, and decreases in *Faecalibacterium*; and the vaginal microbiota increases in *Lactobacillus* species that gradually decline in presence postpartum (90, 91). Beta-diversity, the differences in microbial composition between samples, increases in the third trimester compared to the first trimester (90, 92). *Streptococcus, Lactobacillus*, and *Enterococcus* are enriched during the third trimester, and *Streptococcus* abundance is still enhanced 1 month postpartum, but the abundance of *Faecalibacterium*, which produces butyrate and promotes an anti-inflammatory environment, is reduced (90). Proteobacteria are associated with increased inflammation and transferring the human gut microbiota of the first and third trimester to germ-free mice induced a higher inflammatory response (increases in IFN-γ, IL-2, IL-6, and TNF-α) in mice receiving the third trimester gut microbiota (90). Studies in German Shepherd dogs revealed that bacterial communities shift after giving birth; Fusobacteriae and Bacteroidete*s* decreased while Firmicutes, especially Lactobacillaceae increased (93). It has been suggested that gut microbes have the potential to colonize the vagina or spread by translocation across the epithelium into the blood stream, and this dissemination has the potential to impact pregnancy outcomes (94).

Several probiotics for maternity have appeared on the market while the research on the effect of probiotics on maternity and infants remains slim. How maternal probiotics modulate changes to the gut microbiota that occur during pregnancy must be studied. Probiotics do not, however, significantly alter the vaginal microbiota during pregnancy (95). Neither do they have a significant impact on glycemia or health of offspring in obese pregnancy (96), however a more comprehensive study including a longer probiotic administration period promises to be more revealing (97). Also, the addition of dietary counseling to probiotic consumption of *Lactobacillus rhamnosus* and *Bifidobacterium lactis* in pregnant women with metabolic disorder decreased fasting blood glucose, improved glucose tolerance, and decreased the frequency of gestational diabetes mellitus (98, 99). Although probiotics have been proposed to enhance the integrity of the gut epithelium barrier, studies in overweight pregnant women revealed that probiotics consisting of *Bifidobacterium animals ssp lactis* and *Lactobacillus rhamnosus* failed to reduce the increases in levels of serum zonulin and lipopolysaccharide (LPS) associated with pregnancy (100), both of which suggest impaired intestinal barrier function. Furthermore, infection is an important factor in pre-term labor and pathogenic microbes can be outcompeted by probiotics, yet, according to a systematic review by Jarde et al. (101) probiotics neither increased nor decreased pre-term birth (101). Kriss et al. (102) corroborates this conclusion, but suggests that high probiotic yogurt consumption may be associated with reduction in preterm delivery in Mexican women of normal weight as opposed to overweight (102). Altogether, limited evidence exists that maternal probiotics beneficially impact the mother-infant interface and more research is needed on how changes in the gut microbiota that occur during pregnancy might be modulated by probiotic treatment.

### The immune system during pregnancy

The immune system undergoes hormone related changes during pregnancy to support and tolerate the developing fetus. Prior to our current understanding, it was believed that the mother's immune system was inactivated (103). In the 1980's it was believed that the overwhelming immune environment of the uterus was anti-inflammatory, or resembling a Th2 (high IL-4 and IL-10) response. However, current research testifies to a coordinated pro- and anti-inflammatory response. Characteristic phases mark the progression of pregnancy, starting with an inflammatory phase during implantation with high levels of the cytokines IL-6, IL-8, and TNF-α (103, 104). This pro-inflammatory state is critical for the blastocyst to rupture the uterus epithelial layer and for subsequent tissue repair (103, 104). The second phase is predominantly anti-inflammatory with markedly higher immune tolerance to facilitate rapid growth of the fetus (103, 104). In the third and final stage, pro-inflammatory immune responses are part of promoting contraction and rejection of the placenta (103, 104).

The composition of the uterine immune system during normal pregnancy is dominated by uterine natural killer (uNK) cells which aid in trophoblast invasion of the uterus (104–106) and are tolerogenic in the uterus and pro-fetus, supposedly due to a high presence of IL-10 (106). However, when these cells are relocated to non-fetal environment they resume their cytotoxic function (106). uNK cells expresses the surface marker phenotype of cytokine-producing NK cells (CD56^high^CD16^−^), but are similar to cytotoxic NK cells (CD56^low/−^CD16^+^) and differ from cytokine-producing NK cells circulating in the blood due to the presence of granules (107). In 2005, NK and NKT cells were proposed to fall into different subsets based on Th1- and Th2- like cytokine profiles (108). The NK1/NKT1 subtype produces IFN-γ and the NK2/NKT2 subtype produces IL-5 and IL-13. Furthermore, studies differentiating these subtypes showed decreases in the ratio between NK1/NKT1 and NK2/NKT2 cells were associated with healthy pregnancy (109).

Macrophages are the most abundant antigen presenting cell (APC) type, and, next to NK cells, they are the second most abundant leukocytes (105). Uterine macrophages are similar to M2 macrophages, meaning their phenotype is selective for tissue repair and is highly associated with a classical Th2 response and, like uterine DCs (uDCs), express IL-10, TGF-β, and indolamine 2,3-dioxygenase (IDO) (106). However, unlike M2 macrophages, uterine macrophages are not induced by Th2 cytokines but by macrophage colony-stimulating factor (M-CSF) and IL-10 (107). Uterine macrophages, uNK, and uDCs all play a key role in decidual formation (104). Uterine macrophages and uNK cells also facilitate remodeling of vasculature, especially spiral arteries, transient arteries that supply blood to the endometrium and decidua, through expression of molecular compounds important for angiogenesis (107, 110).

In regard to the adaptive immune system, CD8^+^ T cells are more abundant in the pregnant uterus than CD4^+^ T cells (105). However, the populations of helper CD4^+^ T cells undergo dynamic modulation during pregnancy (105). For one, a Th2-like immune profile dominates over Th1, and, in fact, over-expression of the cytokines associated with Th1, IFN-γ and IL-2, leads to spontaneous abortion (105). Levels of Th17 cells rise in the decidua and peripheral blood during the first trimester, and, by the third trimester, fall back to normal levels (111). While an unbalanced Th17 response is associated with pathological conditions including autoimmune diseases, increases in Th17 cells during early pregnancy may be necessary for the events that take place at this time, like the invasion of trophoblasts (105, 111, 112). CD4^+^CD25^+^ Treg cells also increase during the first trimester, but unlike their Th17 cousins, Treg levels peak during the second trimester in the decidua and peripheral blood (105, 111, 112). Treg cells stimulate uDCs to express IDO, which inhibits T cell proliferation and is also expressed in trophoblasts and macrophages (113). The mechanism of IDO activity is by catalyzing the conversion of tryptophan to kynurenine, an agonist for aryl hydrocarbon receptor (AhR) (114, 115). AhR has been reported to have immunosuppressive and immune-modulatory activity through the induction of TGF-β, IL-10, and IL-22 (114, 116).

Our knowledge is limited on how maternally administered probiotics interact in an immunomodulatory way with pregnancy-associated changes to the immune system. However, the probiotic bacterium *Lactobacillus reuteri* can catabolize tryptophan to indole-3-aldehyde (IAld) under high tryptophan conditions when sugars are depleted as an energy source, and, like kynurenine, IAld is another agonist of AhR (114, 115). Probiotics may therefore be able to enhance the immunosuppressive environment of the uterus either through direct colonization of the placenta and amniotic fluid, or by a peripheral interaction. The mechanisms by which bacteria affect the prenatal environment are further discussed in following sections. Of the few clinical studies in this area, Rautava et al. (117) found that expression of TLR1, TLR7, and MD-2, a surface protein that associates with TLR4 to confer a signaling response to LPS, were decreased in the placenta of mothers receiving *B. lactis*, but TLR3 expression increased. A combination of *B. lactis* and LGG decreased the transcript levels of TLR1, MD-2, and TIR domain-containing adaptor protein (TIRAP), an adaptor protein that connects TLR4 and TLR2 with MyD88 (117). Furthermore, post-delivery treatment with fermented milk with *L. casei* DN11201 increased NK cell levels in the blood and, only at 10 days postpartum, increased IgG4 levels, but the treatment failed to significantly alter the numbers of T and B lymphocytes as well as the ratio of Th1 to Th2 cells and related cytokine levels (118). At 45 days postpartum, breastmilk had significantly increased levels of IL-10 and TNF-α in the group receiving fermented milk, but levels of TGF-β, IL-1, IL-6, IL-8, IL-12, and IgA were unchanged (118). However, VSL#3—a mixture of *Lactobacillus, Bifidobacterium*, and one *Streptococcus* species significantly reduced the decrease of Th2-like cytokines, IL-4 and IL-10 (95). In summary, the tolerogenic properties of probiotics may enhance the tolerogenic maternal state, but it is unclear whether they inhibit the necessary pro-inflammatory stages of pregnancy.

### Pregnancy and autoimmunity

Pregnancy is a contentious topic when it comes to autoimmune disease, especially since many autoimmune diseases, include SLE, RA, and systemic scleroderma, are female-biased (119, 120). These three aforementioned autoimmune diseases are particularly involved with pathogenesis of various kinds of kidney disease, and the kidney is a particularly vulnerable organ during the stress of pregnancy (119). The current recommendation for women with autoimmune disease is to avoid pregnancy, especially in the case of kidney disease, because it may lead to infertility and complications for the pregnant woman and her child (121–126). However, recent evidence shows that serum creatinine and glomerular filtration rate were significantly improved in pregnant patients with lupus nephritis compared to non-pregnant lupus nephritis patients (127). Pregnancy also failed to significantly exacerbate renal flares compared to control patients in lupus nephritis patients (127). Furthermore, pregnancy has been found to dampen symptoms of autoimmune diseases, such as SLE and MS, that are pathologically dependent on imbalanced Th1 and Th17 responses (113). Thus, there is conflicting evidence on how pregnancy impacts autoimmune disease and the current recommendation for women with autoimmune disease is to avoid pregnancy.

While we know that both pregnancy and probiotics may dampen autoimmune disease, the interaction between probiotics and pregnancy in autoimmune disease is poorly understood. It is therefore critical that we seek to understand how the autoimmune ameliorative effects of probiotics impact pregnancies associated with autoimmune disorders.

## Effect of maternal probiotics on offspring

### Neonatal development of the gut microbiota

**Figure 1:**
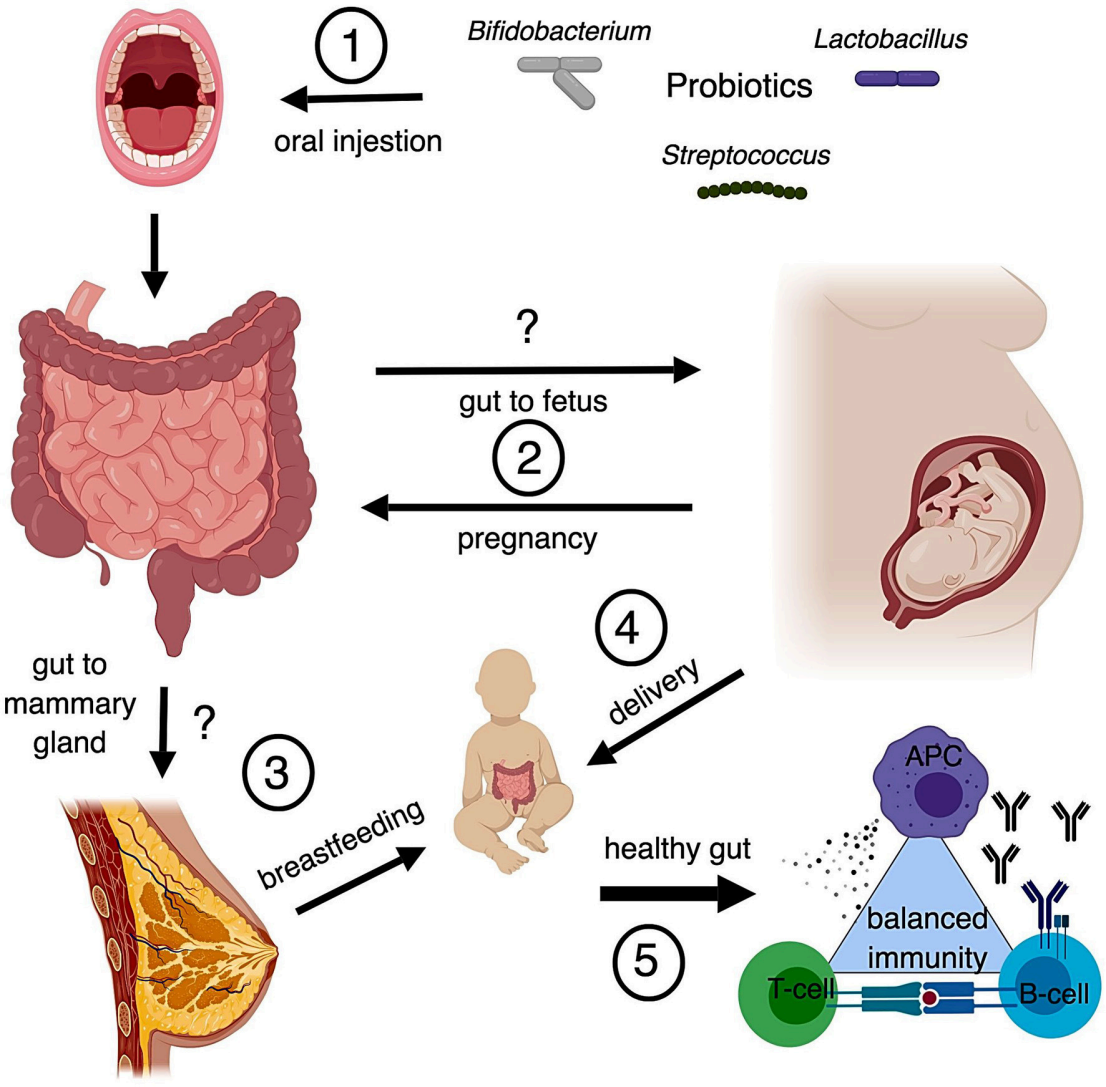
Possible routes of maternal probiotics to neonate (1) Common probiotics include bacteria from the genera *Bifidobacterium, Streptococcus*, and *Lactobacillus*. Oral probiotics modulate populations of bacteria in the gut. (2) Translocation of bacteria from the gut to the fetal environment is controversial, however there is also the possibility that metabolites derived from gut bacteria can influence fetal development as well. While there are known changes to the gut microbiota that occur with pregnancy, the modulation of gut microbiota by probiotics during pregnancy is understudied. (3) Translocation of bacteria from the gut to the mammary gland is also controversial. Studies suggest similarities in the microbial composition between the two and that breastfeeding provides important immunological protection through maternal antibodies and microbes. (4) The method of delivery impacts the development of the neonatal gut microbiome. Infants receive microbiota from their mother's vagina unless they are delivered by C section. Changes to vaginal microbiota from probiotic treatment is controversial. (5) Development of the infant's gut microbiota is critical to forming and maintaining a balanced immune system. Disruption of gut microbiota early in life, through C section or antibiotics, may be linked to autoimmunity and hyperallergenic responses.

The fetus, formerly believed to be sterile, was found to be colonized by bacteria via the placenta and amniotic fluid through groundbreaking research conducted in the early 2010's (128, 129). Up until this point, it was believed that infants' microbiota began with bacteria from their mothers' vaginal canal (130). The discovery that smashed the sterile womb hypothesis was followed by several ideas on how early colonization of the fetal gastrointestinal tract may occur including swallowing amniotic fluid (131), or translocation from a pregnant mothers' gastrointestinal tract to the mesenteric lymph nodes (MLN) and to the mammary glands by phagocytic immune cells (132). Similarities in bacterial load were found between infant feces and the feces, milk, and blood of the mom (132). Supporting this hypothesis, the bacterial load of MLNs in mice was determined to be higher during late pregnancy followed by a steep decline after birth, concomitant with a sharp increase of bacteria present in mammary tissue during lactation (132). Additionally, hormonal changes may increase permeability of the gut epithelium during pregnancy and lactation, further facilitating transfer of bacteria from the gut to the mammary tissue (133). Two studies support a transfer system from the gut to the mammary gland: one showed genetically labeled *Enterococcus faecium* in the meconium of pups born to mice orally gavaged with the bacteria (134), and the other, a more recent study, traced the tissues that fluorescently labeled *Lactobacillus lactis* and *L. salivarius* translocated to when administered via gavage to pregnant BALB/c mice (135). Are these translocated bacteria artifacts of active phagocytic cells or is there an alternative mechanism of transport? While this question remains unanswered, some evidence contradicts the hypothesis of translocation from the gut through the MLN to the mammary glands. For example, Treven et al. (35) conducted a study in which pregnant mice gavaged with 4 × 10^8^ colony forming units (cfu) per dose of either LGG or *Lactobacillus gasseri* K7 (LK7) were sacrificed at 3 and 8 days postpartum. Bacteria from the MLN and mammary gland were assessed. Neither LGG nor LK7 were found in the probiotic treated MLN or mammary gland, yet bacteria from the genus *Lactobacilliceae* were only present in the mammary gland of probiotic treated mice (35). Similarly, treatment of women with VLS#3 during pregnancy showed that the probiotic organisms administered did not enter the mammary gland, but the probiotic group had higher numbers of *Lactobacilli* and *Bifidobacteria* detected in the colostrum of milk (136). However, this contradiction may be explained if translocation is a transient phenomenon, as Perez reported his findings from 1 day postpartum and Treven et al. (35) reported his findings from 3 to 8 days postpartum. In summary, there is evidence that infants receive their microbiota, both through prenatal exposure and through nursing, from the bacteria that colonize the mother's gastrointestinal tract (Figure 1). However, the evidence is not conclusive. As previously mentioned, probiotics were also found to alter the vaginal microbiota, and, therefore, influence the development of the infant's gut microbiota.

With this in mind, literature also supports that the gut microbiota of vaginally delivered newborns are closer in resemblance to the mother's vaginal microbiota than her gut, and newborns delivered by Cesarean section have a microbiota most similar to the skin microbiota (130, 137). In general, the microbiota of an infant lacks the diversity of an adult (93), and the infant's microbiota is most similar to their mother's microbiota at 4 years old (90). It does not reach full diversity until after 4 years of age (138). One study showed the probiotic *L. casei subsp. rhamnosus* administered to infants from birth to 6 months greatly altered the gut microbiota, significantly increasing 682 taxa including other lactic acid bacterial species (139). Proteobacteria represented 60% of the taxa that were significantly increased (139). Another study on the long-term effects of probiotics on atopic children showed that probiotics did not significantly alter the diversity of bacterial phyla and that the gut microbiota is most unstable during the first two years of life with rather dramatic fluctuation in major phyla (140). Also, there was no long-term effect of probiotic treatment on the composition of the gut microbiota (140). However, probiotics did result in the removal of *Lactococcus* from the probiotic treated group during intervention (140). Another study showed similar results, the composition of the gut microbiota changed dramatically over time but retained a notable number of bacterial species from birth to 3 years of age (141). Also, the functional metabolic pathways remained static even though the bacterial communities changed (141). Other studies, such as a placebo-controlled study in Finland and Germany, found that treating mothers with probiotics during lactation significantly altered populations of bacteria, specifically *L. rhamnosus* and *B. longum* given to Finnish women was reflected in the offspring by higher percentages of *Lactobacillus* and *Enterococcus* and lower levels of *Bifidobacterium* (142). Interestingly, the effects varied between the two countries due to differences in the way that infants were fed and seeding microbiota (142). In mice, the colonic gut microbiota was examined with treatment of probiotic fermented milk with *L. casei* DN-114001 (143). This study included four groups: no treatment, only pups received probiotics, only dams received probiotics, and both dams and pups received probiotics (143). *Bifidobacteria* and *Enterobacteria* levels were significantly increased in both groups where probiotics were fed to dams (143). After weaning, groups that did not feed probiotics to the pups saw a decline in *Bifidobacteria* and *Enterobacteria* levels (143). Similarly, maternal treatment with *Clostridium butyricum* increased levels of *Lactobacillus* and *Bifidobacterium* in pups while decreasing levels of *Enterobacter* spp (144). However, after weaning, the group giving *C. butyricum* only to pups overtook all other groups in the levels of *Bifidobacterium* and *Lactobacillus* (144). In all, the infant gut microbiota may be more susceptible to changes in structure, but not function, than the adult gut, and therefore easily influenced by probiotic intervention.

The health implications for either maternally administered or early childhood probiotics are diverse. *L. rhamnosus* when administered at 10^10^ cfu every day for 4 weeks before birth and then continued for 6 months either to breastfeeding mothers or in water given to offspring moderated excessive weight gain in offspring (145). Furthermore, one of the most significant advances in infant probiotic therapy is the mounting evidence that they can reduce the incidence of necrotizing enterocolitis in pre-term infants (131, 146). Besides development of a robust immune system, the infant gut microbiota is critical for angiogenesis in the gastrointestinal system and pre-term infants have a severely deficient microbiota infants (131, 146). Necrotizing enterocolitis is a condition in which the vasculature of the gastrointestinal tract is not well developed, as occurs in pre-term infants, and results in the death of gut tissue infants (131, 146). Studies in germ-free mice show that development of vasculature can be induced by colonizing the gut with normal microbiota, and that the gut epithelium is an important mediator between the microbiota and vasculature (147). Thus, the use of early probiotic intervention has made significant advances in developmental healthcare strategies, with the caveat that perinatal probiotic exposure can alter off-target developmental processes (148).

### Modulation of the developing immune system

At birth, the immune system is naïve and the adaptive immune system is undeveloped. Specific differences exist in a neonatal innate immune system compared to an adult (149). Similar to the uterine environment, newborns have a higher number of NK cells than adults, and the NK cells are deficient in cytotoxic capacities, much like uNK cells (149). Neonatal blood contains more monocytes but less plasmacytoid DCs (pDCs) than adults (150). Additionally, neonatal whole blood monocytes, conventional DCs (cDCs), and pDCs are less responsive and less polyfunctional than their adult counterparts. Interestingly, these differences are most profound following TLR1/2, TLR4, and TLR7/8 stimulation and, for the monocytes and cDCs, only observed in umbilical cord whole blood—a proxy for neonatal blood—instead of PBMC isolated cells (150). This indicates a role for soluble factors that suppressed the responsiveness (151). In general, neonatal immune cells produced less type 1 cytokines, more Th17-inducing cytokines, and more IL-10 (150). As for the adaptive immune system, like the uterine environment, Th1 cytokines such as IFN-γ are reduced (152). Studies in rats showed that germinal centers in the spleen, lymph nodes, and Peyer's patches didn't develop until after 20 days of age, which is between the times when rats are weaned and reach sexual maturity (153). Interestingly, nascent follicles begin to form around seven days of age, which is about 5 days before rats begin to nibble on solid food (153). The beginning of eating a non-maternally supplemented diet marks the maturation of the immune system adaptive compartments and the end of provisional protection from a mother's milk. Prior to maturation, maternal antibodies are transferred to infants through milk and constitute early adaptive immunity (154). Specifically, IgA is the main antibody produced by the mucosal associated lymphoid tissue (MALT) of the mammary glands (154). IgA crosses the epithelium by binding to polymeric immunoglobulin receptor (pIgR) on the basal side of epithelial cells and transcytosis into the milk ducts (154). Early endogenous mucosal IgA production can be stimulated earlier in the absence of maternally transferred antibodies, such as the case when immunocompetent mice nurse on immunocompromised mothers (155). In this scenario, it is still unknown whether or not there is an immune stimulatory factor in milk that enhances early endogenous IgA production. However, it is known that the maternally transferred microbiota are responsible for jumpstarting the immune system (156). Translocation of bacteria from the gut to the MLN, where antigens from the bacteria are presented to educate adaptive immune cells, occurs spontaneously in infants during the nursing period (157). Therefore, the gut microbiota is important for education and maturation of the immune system.

Perinatal probiotics can shape the development of the immune system. Maternally administered *B. lactis* reduced TLR7 mRNA expression in the fetal intestines, whereas a combination of LGG and *B. lactis* decreased TLR6 mRNA expression (117). Postnatal probiotic supplemented food given to lactating mice significantly increased their offspring's Treg cell populations in saline control and peanut extract induced allergic response groups, but did not change inducible Treg cell populations (158). Probiotic treatment also decreased IL-6, IL-9, TNF-α, IL-17, and IL-7 mRNA expression in the pups' spleens (158). When probiotic fermented milk containing *L. casei* DN-114001 was administered to dams, the offspring had in increased level of secretory IgA in intestinal luminal fluid at 12 days of age, however at 28 days of age the same pups had a lower number of cells producing IgA in the small intestines (143). Additionally, when dams were fed probiotics their offspring had decreased numbers of macrophages at 12 days of age, but when probiotics were administered to postnatally the number of mature F4/80^+^ macrophages was significantly increased by 45 days of age (143). Contrary to these findings, *C. butyricum* treatment in either dams or pups, although it similarly increased levels of *Bifidobacterium*, did not significantly affect the levels of secretory IgA in intestinal fluid at 14 and 28 days of age (144). Probiotic milk containing a combination of 5 × 10^10^ cfu *L. rhamnosus*, 5 × 10^10^ cfu *B. animalis*, and 5 × 10^8^ cfu *L. acidophilus* per dose administered daily during pregnancy conferred a decreased risk of atopic dermatitis in infants and a decrease in the proportion Th22 cells, however no difference in other T helper cell subsets or Th1 to Th2 ratio was observed (159). *B. pseudocatenulatum* administered 2 days postnatally to C57BL/6 mice until 21 days old decreased stress hormones in a model for gut-brain involvement in early life trauma and chronic stress induced by maternal separation (160). The probiotic treatment also dampened LPS induced IFN-γ expression in MLN cells, which attributed to increased sensitivity to glucocorticoids (160). Altogether, probiotics received perinatally encourage endogenous adaptive immune response, enhance immune system maturation, and reduce pro-inflammatory immune responses. Yet, these results are still controversial and many discrepancies exist.

### Influence of maternal probiotics on autoimmunity in offspring

While maternal probiotics have attracted attention for the health of newborns by reducing allergies and incidence of pre-eclampsia (161, 162), only one study has been has been reported on perinatal probiotics for autoimmune disease. This study found no association between incidence of celiac disease in children and maternally administered probiotics during late pregnancy and nursing (163). Although there is scant evidence for maternal probiotic intervention for autoimmune disease in infants, there is evidence that early microbial associated disruptions may be a contributing factor to the development of autoimmune disease. In children who develop type 1 diabetes, a decrease in the diversity of the gut microbiota occurs after seroconversion and before diagnosis (141). The bacterial genera *Blautia* and *Ruminococcus* were found in higher abundance in children who developed type 1 diabetes and contributed to increased serum levels of branched-chain amino acids (141). Furthermore, the genetic expression of the microbiota changes before the onset of clinical symptoms, and these changes include increases in sugar transport systems (141). Also, children born by C-section or exposed to prenatal antibiotics had increased risk of celiac disease, asthma and type 1 diabetes (164). Thus, evidence supports the importance of maternal transfer of the gut microbiota in prevention of autoimmune disease and creates a gap that could potentially be filled by probiotics intervention.

## Conclusions

Significant grounds have been covered in establishing the link between dysbiosis and autoimmune disease marked by the presence of a leaky gut and systemic inflammation. From what we can study of the complex ecosystem that is the gut microbiota, probiotics induce significant changes to the normal microbiota and enhance anti-inflammatory immune reactions. Through their immunological modulatory capacity, probiotics can therefore alleviate autoimmune disease severity.

The normal gut microenvironment undergoes specific changes during maternity including an increase in gut barrier permeability. Furthermore, specific changes in the immune system occur to support and tolerate the developing fetus. Maternally administered probiotics may impact the health of a child by altering the bacteria that initially colonize the developing infant. Maternal probiotics thus have the potential to influence neonatal immune development since the initial programming of the immune system is dependent on the gut microbiota.

As the influence of the maternal-neonatal interface on the gut microbiota in autoimmune disease is a poorly understood, further research is required regarding the impact of maternal probiotics on the development of the fetus and the implications in autoimmune disease. A number of gaps exist in the literature regarding maternal probiotics for autoimmune diseases in both mothers and their children. Designing studies aimed at understanding how probiotics act in an immunomodulatory capacity to ameliorate or exacerbate autoimmune diseases in pregnancy and infancy is, without excuse, a requisite edge for cutting into the issues surrounding autoimmunity and pregnancy.

## Author contributions

BS: research, writing, editing, figure design; XL: research, editing.

### Conflict of interest statement

The authors declare that the research was conducted in the absence of any commercial or financial relationships that could be construed as a potential conflict of interest.
